# Thyroid dysfunction in Hashimoto’s thyroiditis: a pilot study on the putative role of miR-29a and TGFβ1

**DOI:** 10.1007/s12020-024-03965-3

**Published:** 2024-07-17

**Authors:** Maria Consiglia Trotta, Daniela Esposito, Raffaela Carotenuto, Rosa di Fraia, Lucia Digitale Selvaggio, Francesca Allosso, Marina Russo, Giacomo Accardo, Roberto Alfano, Michele D’Amico, Daniela Pasquali

**Affiliations:** 1 Department of Experimental Medicine, University of Campania “Luigi Vanvitelli”, Naples, Italy; 2 Department of Internal Medicine and Clinical Nutrition, Institute of Medicine, Sahlgrenska Academy, University of Gothenburg, Gothenburg, Sweden; 3 Department of Endocrinology, Sahlgrenska University Hospital, Gothenburg, Sweden; 4 Department of Advanced Medical and Surgical Sciences, University of Campania, “Luigi Vanvitelli”, Naples, Italy; 5 PhD Course in National Interest in Public Administration and Innovation for Disability and Social Inclusion, Department of Mental, Physical Health and Preventive Medicine, University of Campania “Luigi Vanvitelli”, Naples, Italy; 6 School of Pharmacology and Clinical Toxicology, University of Campania “Luigi Vanvitelli”, 80138 Naples, Italy; 7 Azienda Sanitaria Locale Napoli 3 Sud, Naples, Italy

**Keywords:** Hashimoto Thyroiditis, miR-29a, TGF-β1, TSH, Hypothyroidism, Euthyroidism

## Abstract

**Purpose:**

Hashimoto’s thyroiditis (HT) is one of the most common causes of thyroid dysfunction in iodine sufficient worldwide areas, but its molecular mechanisms are not completely understood. To this regard, this study aimed to assess serum levels of miRNA-29a (miR-29a) and transforming growth factor beta 1 (TGFβ1) in HT patients with different patterns of thyroid function.

**Methods:**

A total of 29 HT patients, with a median age of 52 years (21–68) were included. Of these, 13 had normal thyroid function (Eu-HT); 8 had non-treated hypothyroidism (Hypo-HT); 8 had hypothyroidism on replacement therapy with LT4 (subst-HT). All patients had serum miR-29a assayed through qRT-PCR and serum TGFβ1 assayed by ELISA.

**Results:**

Serum miR-29a levels were significantly down-regulated in patients with Hypo-HT compared to Eu-HT patients (P < 0.01) and subst-HT patients (P < 0.05). A significant negative correlation was detected between serum miR-29a levels and TSH levels (r = −0.60, P < 0.01). Serum TGFβ1 levels were significantly higher in Hypo-HT than both Eu-HT (P < 0.01) and subst-HT patients (P < 0.05). A negative correlation was observed between serum miR-29a and TGFβ1 (r = −0.75, P < 0.01).

**Conclusions:**

In conclusion, Hypo-HT patients had lower levels of serum miR-29a and higher levels of TGFβ1 in comparison with Eu-HT patients. Worthy of note, subst-HT patients showed restored serum miR-29a levels compared with Hypo-HT group, associated with lower serum TGFβ1. These novel findings may suggest a possible impact of replacement therapy with levothyroxine on serum miR-29a levels in HT.

## Introduction

Hashimoto’s thyroiditis (HT) is one of the most common causes of hypothyroidism in iodine sufficient worldwide areas. The pathogenesis of this autoimmune disorder is due to a combination of genetic and environmental factors [1]. HT is characterized by autoimmune-related destruction of thyroid epithelial cells that may result in thyroid dysfunction. Cellular and humoral immunity, such as chemokines and cytokines, have a key role in the development of HT [2]. Almost all patients show high levels of thyroid peroxidase and thyroglobulin antibodies, whose presence is critical for clinical management [3]. Clinical features of HT vary greatly, from asymptomatic disease to clinical hypothyroidism. In some cases, normal thyroid function is preserved, despite antibodies’ presence [4, 5].

Available data suggest that transmission of molecules, as microRNAs (miRNAs), interfering with RNA of recipient cells, could affect the biological activity of cells [6, 7] and have an impact on HT [8–12]. miRNAs are a class of non-coding RNA molecules with 18–22 nucleotides, which interaction with mRNA target could prevent protein translation, resulting in mRNA degradation or translational repression [13]. miRNAs have been implicated in various cellular processes such as differentiation, proliferation and apoptosis. Recent studies have shown that miRNAs play a crucial role in the regulation of immune response and inflammation, and dysregulation of miRNA expression has been linked to autoimmune diseases, including HT [14]. Among the wide scenario of miRNAs involved in biological controls, miR-29a has been identified differentially expressed in HT patients with eu- and hypothyroidism [15]. This miRNA belongs to the miR-29 family, composed of miR-29a, b-1, b-2 and c cluster [16]. It has been previously shown that down-regulation of miR-29a contributes to the development of autoimmune disorders [16–25]. In addition, available data suggest that miR-29a is involved in the modulation of T-cells [15, 16], which are responsible for the chronic inflammatory underlying the evolution from euthyroidism to hypothyroidism in HT patients [15].

miR-29 family has also shown strong anti-fibrotic properties [26], supported by the evidence of a crass-talk between miR-29 and transforming growth factor beta (TGFβ) pathway [27–29]. The TGFβ pathway is a key signaling pathway involved in fibrosis. TGFβ promotes the differentiation of fibroblasts into myofibroblasts, which produce excessive extracellular matrix leading to fibrosis. Worthy of note, alterations of TGFβ1 expression have been associated to HT [30, 31], with available data suggesting that thyroid hormones may inhibit TGFβ signaling [32].

Our hypothesis is that different expressions of miR-29a and TGFβ1 in patients with HT may play a role in impairment of thyroid function. Therefore, this study aimed to assess serum levels of miR-29a and TGFβ1 in HT hypothyroid patients in comparison with euthyroid patients as well as hypothyroid patients on replacement therapy with levothyroxine (LT4).

## Methods

### Clinical design

The study was approved by Ethical Committee of University of Campania “Luigi Vanvitelli” (protocol number 0028416/i, 23/11/2020) and was performed according to the Declaration of Helsinki and Good Clinical Practice guidelines. A total of 29 HT patients attending the Unit of Endocrinology (University of Campania “Luigi Vanvitelli”) were enrolled. Inclusion criteria were age 20–68, positive levels of anti-thyroid peroxidase (anti-TPO) or anti-thyroglobulin (anti-TG) antibodies and signed informed written consent. Exclusion criteria were: medical history of thyroid malignancy or hyperthyroidism; medical treatment affecting thyroid function; pregnancy within the last 12 months prior to the initial diagnosis of HT; cancer history; smoking, obesity (body mass index >30 kg / m2); depressive and / or anxious disorders; other clinically evident autoimmune diseases (rheumatic arthritis, systemic lupus erythematosus, systemic sclerosis, mixed connective tissue disease, scleroderma, inflammatory myositis, dermatomyositis, polymyositis, vasculitis, psoriatic arthritis, ankylosing spondylitis, post-infectious arthritis, non-seronegative spondylitis).

### Serum miR-29a assessment

Total RNA, including small RNAs was extracted from serum samples, stored at −20 °C, by using MiRneasy serum / Qiagen plasma kit. To monitor the efficiency of miRNA extraction, the synthetic miRNA Syn-cel-miR-39 (miScripit miRNA Mimic 5 nM) was added to each sample before its processing. The concentration and purity of isolated RNA was evaluated by NanoDrop spectrometry. The mature miRNAs were converted to cDNA (MiScript II Reverse Transcription Kit, Qiagen), then serum miR-29a was amplified by CFX96 Touch TM Real-Time PCR Detection System (Biorad), using the MiScript SYBR Green PCR Master Mix (Qiagen) and the appropriate MiScript Primers Specific assay (Qiagen). RT-Real Time PCR (qRT-PCR) data were analyzed according to the relative quantization method of 2^^-ΔΔCT^, by using the Syn-cel-miR-39 as the exogenous control for normalization of the results [13], by considering patients with normal thyroid function as reference group (calibrator)

### Serum TGF-β1 determination

Serum TGF-β1 levels were detected by using Human TGFβ1 (Transforming Growth Factor Beta 1) ELISA Kit (EH0287, FineTest), according to the manufacturer’s protocol.

### Statistical analysis

Continuous variables are presented either as mean (SD) or median (range) and categorical variables as number (%). Data were analyzed by using the Mann-Whitney test. Pearson correlation analysis was performed to analyze the correlation among serum miR-29a and clinical parameters, by reporting the results as Pearson coefficient (r). All results have been considered significant for P < 0.05. Statistical analyses were performed using GraphPad Prism software version 8.0.

## Results

### Characteristic of the patients

A total of 29 patients (4 men, 25 women) with HT were included in the study. Of those, 13 (45%) had normal thyroid function (Eu-HT group), 8 patients (27.5%) had hypothyroidism, and did not receive replacement therapy (Hypo-HT group); 8 patients (27.5%) had hypothyroidism and were on replacement therapy with LT4 at a mean dose of 1.13 µg/kg (subst-HT group) (Table 1).

**Table 1:** Clinical characteristics in patients with normal thyroid function (Eu-HT), non-treated hypothyroidism (Hypo-HT) and hypothyroidism on replacement therapy with LT4 (1.13 µg/kg) (subst-HT)

	Eu-HT (No = 13)	Hypo-HT (No = 8)	subst-HT (No = 8)
**Age,** **(years)**	30 (20–66)	37 (21–53)	52 (21–68)*^,^**
**HT duration (years)**	5.7 (3–13)	2.9 (1–6)	13 (9–17)***
**Female sex (%)**	92%	37.5%	100%
**TSH (uUI/ml)**	2.5 (0.8–4.1)	16.8 (6.3–53.6)*	2.5 (0.6–4.8)***
**FT3 (ng/dl)**	3.1 (2.3–5.4)	1.1 (0.2–4.9)	2.3 (1.1–3.3)
**FT4 (ng/dl)**	1.8 (0.8–9.9)	1.1 (0.5–1.4)	1.6 (1.0–2.8)
**Anti-TG(UI/ml)**	195 (1.3–752)	527 (34–2100)	193 (16–637)
**Anti-TPO (UI/ml)**	172 (89–1300)	650 (9.9–2000)	360 (30–1000)
**Positive family history (%)**	46%	25%	12.5%

Values are reported as median (range) or as percentage (%)

*HT* Hashimoto’s thyroiditis, *TSH* Thyroid-Stimulating Hormone, *FT3* free triiodothyronine, *FT4* free thyroxine, *anti-TG* anti-thyroglobulin antibodies, *anti-TPO* anti-thyroid peroxidase antibodies

*P < 0.01 vs Eu-HT, **P < 0.05 vs Hypo-HT and ***P < 0.01 vs Hypo-HT

The median age was 30 (20–66) years, 37 (21–53) and 52 (35–66), in the Eu-HT, Hypo-HT and subst-HT group, respectively (Hypo-HT vs Eu-HT, P = 0.10; Hypo-HT vs subst-HT, P = 0.02). Median duration of HT was 5.7 (3–13), 2.9 (1–6) and 13 (9–17) years in the three groups respectively (Hypo-HT vs Eu-HT, P = 0.27; Hypo-HT vs subst-HT, P < 0.01) (Table 1).

As expected, Thyroid-Stimulating Hormone (TSH) levels were within the normal range in Eu-HT and in subst-HT group, with a median of 2.5 (0.8–4.1) uUI/ml and 2.5 (0.6–4.8) uUI/ml, respectively. Conversely, TSH levels were significantly increased in Hypo-TH patients with a median of 16.8 (6.3–53.6 uUI/ml; Hypo-HT vs Eu-HT, P < 0.01 and Hypo-HT vs subst-HT, P < 0.01) (Table 1).

### Modulation of serum miR-29a and its correlation with TSH levels

**Fig. 1:**
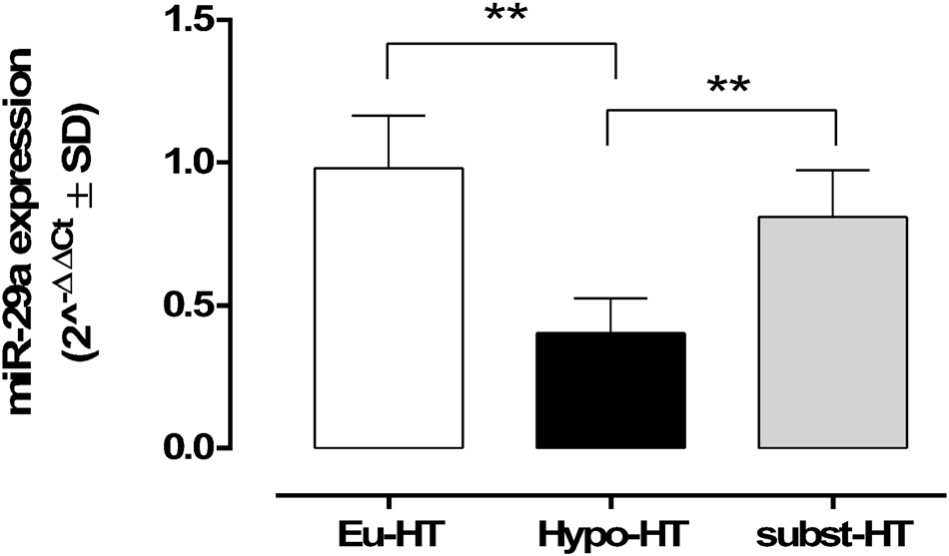
Serum miR-29a in patients with normal thyroid function (Eu-HT), non-treated hypothyroidism (Hypo-HT) and hypothyroidism treated with LT4 (1.13 µg/kg) (subst-HT). miR-29a levels are reported as 2^^−ΔΔCt^ (mean ± SD). **P < 0.01

Serum miR-29a was differentially expressed between the serum samples analyzed. Particularly, serum miR-29a (2^^-ΔΔCt^ ± SD) were 0.98 ± 0.2, 0.40 ± 0.1 and 0.80 ± 0.2 in the Eu-HT, Hypo-HT and subst-HT group, respectively. Serum miR-29a levels were significantly downregulated in the Hypo-HT group in comparison to the Eu-HT (P < 0.01) and to subst-HT group (P < 0.01) (Fig. 1).

**Fig. 2:**
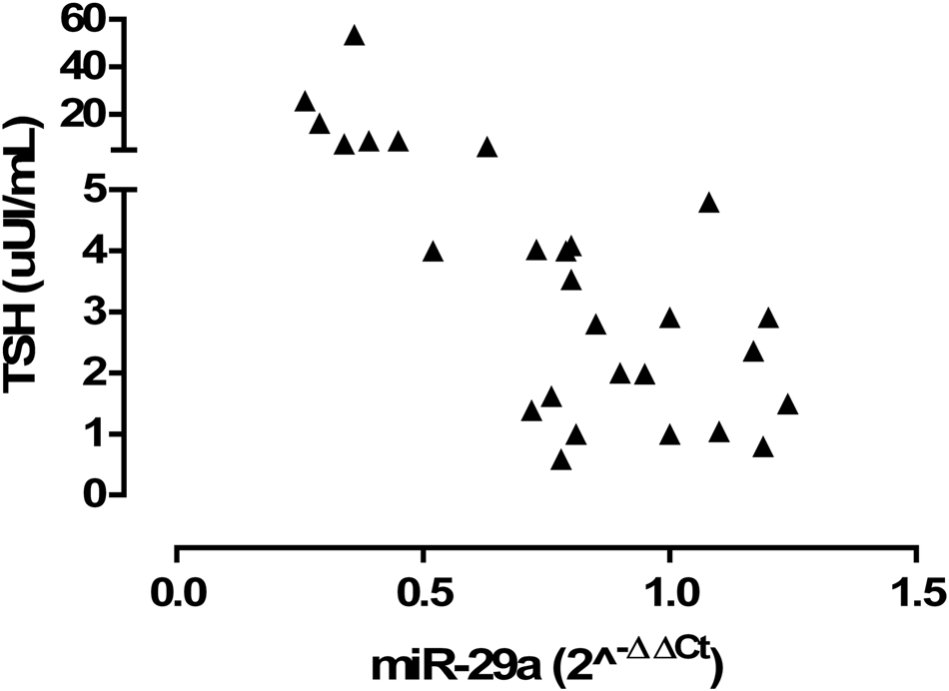
Pearson correlation analysis of serummiR-29a (2^^−ΔΔCt^) with TSH levels (uU/mL); r = −0.60, P < 0.01

Interestingly, a significant negative correlation was detected between serum miR-29a levels and TSH levels (r = −0.60, P < 0.01; Fig. 2).

### Serum TGFβ1 modulation and its correlation with serum miR-29a

**Fig. 3:**
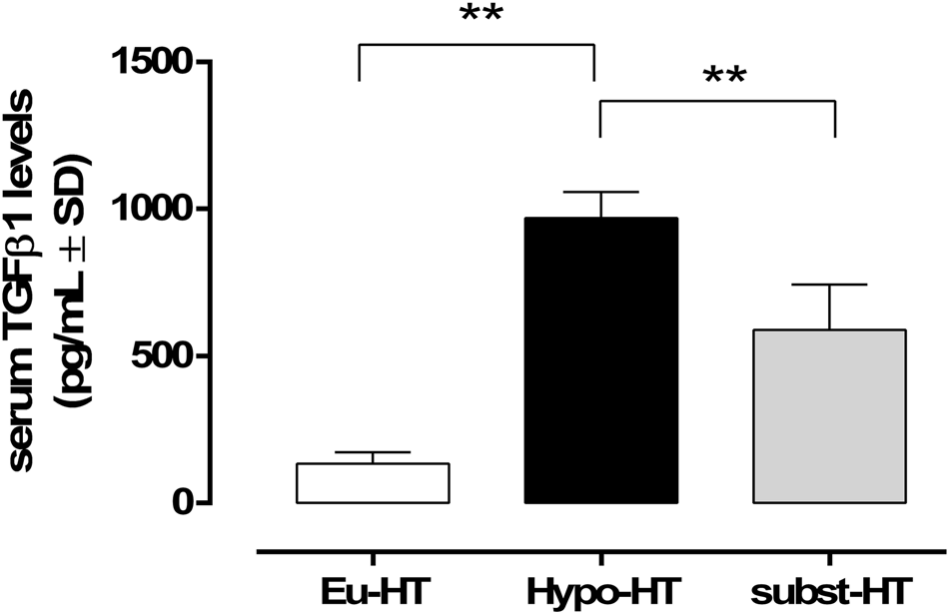
Serum TGFβ1 levels in patients with normal thyroid function (Eu-HT), non-treated hypothyroidism (Hypo-HT) and hypothyroidism treated with replacement therapy LT4 (1.13 µg/kg) (subst-HT). TGFβ1 levels are reported as pg/mL (mean ± SD). **P < 0.01

Serum TGFβ1 levels (pg/mL ± SD) were 134 ± 39, 969 ± 88, 589 ± 153 in the Eu-HT, Hypo-HT and subst-HT group, respectively. The levels of serum TGFβ1 were significantly higher in Hypo-HT in comparison to Eu-HT (P < 0.01) and subst-HT patients (P < 0.01) (Fig. 3).

**Fig. 4:**
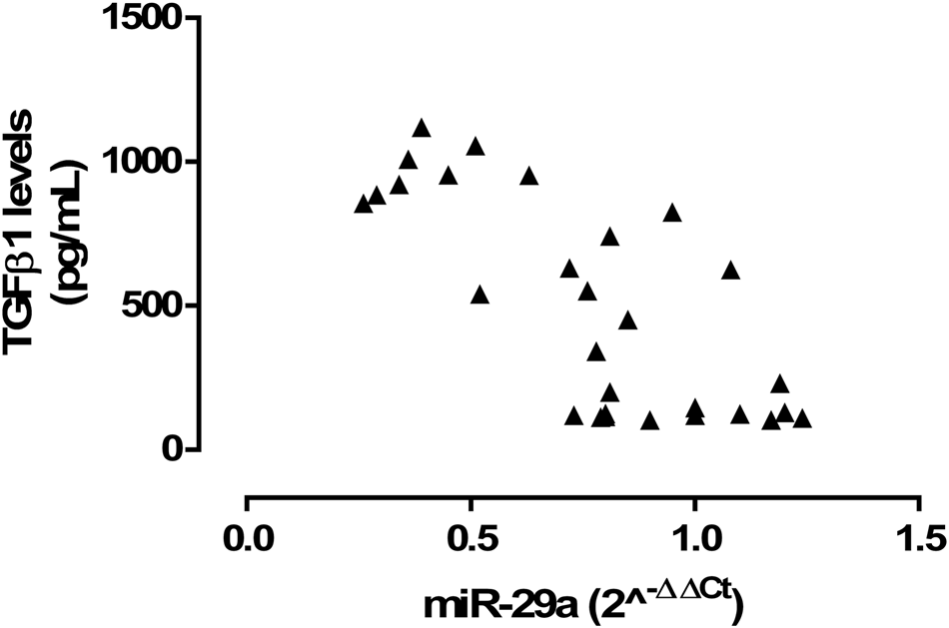
Pearson correlation analysis of serum miR-29a(2^^−ΔΔCt^) with TGFβ1 levels (pg/mL); r = −0.75, P < 0.01

A negative correlation was observed between serum miR-29a and TGFβ1 (r = −0.75, P < 0.01; Fig. 4). In contrast to this, the levels of TSH fitted with the levels of TGFβ1.

## Discussion

HT, a common autoimmune thyroid disorder, is currently considered the leading cause of hypothyroidism in developed countries [33]. HT is caused by autoimmune mediated destruction of thyroid gland and characterized by stromal fibrosis and parenchymal lymphocytes infiltration [33, 34]. Patients with HT may develop thyroid dysfunction. However, in some HT patients, thyroid function is preserved despite the presence of autoantibodies [4, 5].

Although a combination of genetic and environmental factors has been associated with HT development [1], the molecular mechanisms leading to thyroid dysfunction in HT patients are still unclear. Chronic inflammation induced by T-cells is considered as the key factor for the evolution of HT from euthyroidism to hypothyroidism [15]. Specifically, HT patients are characterized by a higher presence of Th17 cells in thyroid gland [33]. These CD4 + T lymphocytes are able to produce the pro-inflammatory interleukin 17 (IL-17) [35] and seem to be associated with several autoimmune disorders [36]. Li et al. showed that serum levels of IL-17 were increased in patients with HT [33]. Interestingly, in that study, serum IL-17 concentration was found to be inversely correlated with residual thyroid function and directly correlated with thyroid fibrosis [33]. HT patients are also characterized by a strict correlation between hypothyroidism occurrence and type-1 activation of CD8( + ) T-cells [37]. These lymphocytes have been considered as important inducers of thyroid epithelial cell fibrosis [38].

The interplay between the pro-fibrotic TGFβ pathway and the pathogenesis of autoimmune thyroid disease has been deeply investigated [39]. TGFβ cytokine superfamily regulates a broad range of essential cellular activities, including proliferation, differentiation, extracellular matrix dynamics and autoimmune processes in many types of cells [40–42]. Specifically, TGFβ is involved in thyroid gland homeostasis and function, by modulating innate and adaptive immunity [39]. TGFβ plays a complex role in the pathogenesis of HT, and its effect can vary depending on the stage of the disease. Of interest, TGFβ can either promote or inhibit thyroid autoimmune disease, and its concentration in the serum can serve as a prognostic marker for the progression of the disease [39]. At the initial stage of HT, TGFβ exerts an autoimmunity-suppressive role [43, 44]. However, as the disease progresses, higher serum concentrations of TGFβ have been found, enhancing intrathyroidal fibrotic activity [38, 39]. In particular, the pro-fibrotic TGFβ1 isoform may contribute to the progression of HT and development of hypothyroidism [30, 31, 45]. In line with previous data, we found that TGFβ1 levels were significantly higher in Hypo-HT in comparison to Eu-HT. Interestingly, TGFβ1 levels were also higher in Hypo-HT in comparison with subst-HT patients, suggesting a possible effect of thyroid hormones on TGFβ1 concentration.

It has been shown that TGFβ family is able to downregulate miR-29 cluster [27, 46–48], that includes miR-29a, miR29b and miR-29c. Available data suggest that these miRNAs have strong anti-fibrotic effects [26] by negatively regulating pro-fibrotic mediators such as collagens, fibrillins, elastin and TGFβ in a loop mechanism [27–29]. Moreover, miR-29 family plays a key role in the autoimmune disorders’ development [17]. Particularly, down-regulated miR-29a expression has been previously associated with several autoimmune disorders, such as myasthenia gravis [18]; arthritis [16]; systemic lupus erythematosus [19]; multiple sclerosis [20]; type I diabetes [21–24] and Crohn’s disease [25].

Recently, differences in miR-29a levels in peripheral blood T cells were found between euthyroid and hypothyroid patients with HT [15]. Therefore, a potential role has been hypothesized for this miRNA in the regulation of T-cells during the evolution from euthyroidism to hypothyroidism in HT [15]. Our study showed for the first time that serum miR-29a levels were higher in HT hypothyroid patients on replacement therapy compared to hypothyroid patients without replacement therapy, suggesting a possible impact of hormone replacement therapy on serum miR-29a levels.

In this study, we show a negative correlation between serum miR-29a and TGFβ1 in HT patients. A previous study has shown that TGFβ1 may lead to the reduction of miR-29a levels in fibroblasts in patients with systemic sclerosis. Similarly, TGFβ1 has been shown to induce significant downregulation of miR-29a in a cell line derived from human proximal tubule cells, leading to an increase in collagen IV. However, no study has reported the relation between serum miR-29a and TGFβ1 in thyroid diseases so far. In line with previous results [30–32], we found that serum TGFβ1 levels were significantly higher in Hypo-HT than Eu-HT patients. However, this is the first study to show a negative correlation between serum miR-29a and TGFβ1 in HT patients.

This crosstalk may suggest that high production of local TGFβ1 could reduce miR-29a expression, leading to increased thyroid fibrosis, inflammation, and HT progression, resulting in progressive thyroid dysfunction. Conversely, the reduction in TGFβ1 levels after hormone replacement therapy could lead to higher miR-29a levels, exerting anti-fibrotic and anti-inflammatory actions. However, our knowledge about the interactions between miR-29a and TGFβ1 is still limited and therefore no conclusion is definitive in HT, especially because of the lack of local tissue evidence and possible pharmacological intervention on miR-29a or TGFβ1.

## Data Availability

The original contributions presented in the study are included in the article, further inquiries can be directed to the corresponding author.
